# Inter- and intra-individual variability of active glucagon-like peptide 1 among healthy adults

**DOI:** 10.15761/jts.1000404

**Published:** 2020-06-16

**Authors:** AC Sylvetsky, V Bauman, J Abdelhadi, JE Blau, KJ Wilkins, KI Rother

**Affiliations:** 1 Diabetes, Endocrinology, and Obesity Branch, NIDDK, National Institutes of Health, Bethesda, MD 20892, USA; 2 Department of Exercise and Nutrition Sciences, Milken Institute School of Public Health, The George Washington University (Washington, DC), USA; 3 Biostatistics Program, Office of the Director, NIDDK, NIH, Bethesda, MD 20892, USA

## Abstract

**Objective:**

To determine whether sex, age, and body mass index are correlated with active glucagon-like-peptide 1 concentrations and to investigate glucagon-like-peptide 1 reproducibility during repeated oral glucose tolerance tests.

**Methods:**

Sixty-one healthy volunteers underwent four 2-hour repeated oral glucose tolerance tests approximately 1 week apart. Because this randomized same-subject crossover trial was designed to investigate effects of non-nutritive sweeteners, participants received 355 mL (12 ounces) of water or a beverage containing non-nutritive sweeteners 10 minutes prior to each oral glucose tolerance test. Blood samples were collected 10 minutes before, and 0, 10, 20, 30, 60, 90, and 120 minutes following ingestion of 75 grams of glucose.

**Results:**

Basal active glucagon-like-peptide 1, peak glucagon-like-peptide 1, and glucagon-like-peptide 1 area-under-the-curve were higher in men than women (all p ≤0.04), adjusting for body mass index and age. Fasting and stimulated active glucagon-like-peptide 1 results were highly reproducible with little within-subject variability (between-subjects to within-subject variability ratio 4.2 and 3.5 for fasting glucagon-like-peptide 1 and glucagon-like-peptide 1 area-under-the-curve).

**Conclusion:**

Men had higher active glucagon-like-peptide 1 concentrations than women. In contrast to considerable inter-individual variability of basal and stimulated active glucagon-like-peptide 1 concentrations, intra-individual variability was low, consistent with tight physiological regulation.

## Introduction

Glucagon-like-petide-1 (GLP-1) is secreted by enteroendocrine L-cells located predominantly in the lower small intestine and colon. GLP-1 has several important biological functions, including slowing of gastric emptying, induction of satiety, stimulation of insulin release, and suppression of glucagon [1]. The half-life of GLP-1 is only 1–2 minutes, as it is rapidly degraded and inactivated by dipeptidyl peptidase 4 (DPP-4) [2]. Individuals with obesity are typically reported to have lower GLP-1 responses to a mixed meal or an oral carbohydrate challenge, compared with lean controls [3,4]. No marked differences have been found between individuals with obesity, with or without type 2 diabetes. Thus, GLP-1 secretion does not appear to play a role in the pathogenesis of type 2 diabetes.

In the past 20 years, the discovery and development of GLP-1 receptor agonists and DPP-4 inhibitors have markedly advanced pharmacological treatment options for obesity and diabetes [5,6], with novel compounds continuing to emerge in the pharmaceutical pipeline [7]. For the design and conduct of clinical trials in this field, it is essential to recognize demographic and anthropometric factors that influence GLP-1 secretion and to be aware of its’ inter- and intra-individual variability. The objective of the present analysis was to examine effects of age, sex, and body mass index (BMI) on fasting and glucose-stimulated active GLP-1 release in healthy volunteers, and to determine the extent to which active GLP-1 responses vary within individuals during serial oral glucose tolerance tests (OGTTs).

## Materials and methods

Sixty-one healthy adults underwent four 75 g OGTTs approximately one week apart, following a 10-hour overnight fast. Subjects were 18 years of age or older, had no known active medical conditions (in particular, no glucose intolerance or diabetes), and were not taking medications (with the exception of oral contraceptives). This is a secondary analysis of data collected during a study designed to investigate metabolic effects of non-nutritive sweeteners (NNS, NCT01200940) and was approved by the Institutional Review Board of the National Institute of Diabetes and Digestive and Kidney Diseases (NIDDK). All subjects provided written informed consent prior to the study procedures.

Because the study was designed to investigate metabolic effects of NNS, participants received 355 mL (12 ounces) of water or a beverage containing NNS 10 minutes prior to each OGTT. The study drinks consisted of commercially available, caffeine-free diet sodas, water with sucralose and/or acesulfame-potassium in concentrations comparable to one of the diet sodas, or water (control). Procedures and results were previously published [8]. Briefly, when diet sodas were ingested prior to the OGTT, active GLP-1 responses were slightly higher compared to water, but gastric emptying and satiety were unaffected [8].

### Measures

Blood samples were obtained 10 minutes prior (−10 min) and 0, 10, 20, 30, 60, 90, and 120 minutes following ingestion of a 75 g oral glucose load. Active GLP-1 was measured by enzyme-linked immunosorbent assay (Millipore, Billerica MA, USA). The lowest detectable level of active GLP-1 was 2 pmol/L (inter-assay CV 8% and intra-assay CV 7%). DPP-IV inhibitor was added to blood collection tubes prior to sample collection for analysis of active GLP-1. Serum glucose was measured using the glucose oxidase method (inter-assay CV 3.9% at 43.2 mg/dL (2.4 mmol/l) and 1.2% at 398.2 mg/dL; intra-assay CV 2.9% at 43.2 mg/dL and 0.4% at 398.2 mg/dL).

### Statistical analysis

Data from the control condition (water ingested 10 minutes prior to OGTT) were used to determine active GLP-1 in relation to age, sex, and BMI. Data from all OGTTs (whether preloads of water, water with NNS, or caffeine-free, diet sodas) were used to evaluate within subjects variability of active GLP-1 serially across the four OGTTs, using linear mixed-effects models with log-transformed values. As the order of the preloads was randomly assigned, we assumed random subject-specific intercepts. A ratio of variance [9] due to between-subject variability (heterogeneity in individual active GLP-1 response) relative to within-subject variability (variability in the same individual across each of the four conditions) was calculated to represent the odds that differences in active GLP-1 were due to inter-individual as opposed to intra-individual variability. Statistical analyses were performed in R, version 3.2.1 (The R Foundation for Statistical Computing) and SAS, version 9.4 (SAS Institute, Cary, NC). Statistical significance was determined as p < 0.05.

## Results

Demographic and anthropometric characteristics, fasting active GLP-1, peak active GLP-1, active GLP-1 AUC, and ratios of inter-individual to intra-individual variability are shown overall and by sex in Table 1. Compared to females, males had higher fasting (6.6 ± 6.1 vs. 3.3 ± 3.4 pmol/L, p=0.01) and peak GLP-1 (18.9 ± 11.9 vs. 13.1 ± 8.4 pmol/L, p=0.03). Active GLP-1 AUC also tended to be higher in males compared to females (1455.0 ± 970.3 vs. 947.9 ± 550.0 pmol/L/120 min, p=0.02). Neither fasting nor peak active GLP-1 concentrations were correlated with age, BMI, fasting insulin, or insulin AUC [data not shown]. Intra-individual basal and peak active GLP-1 concentrations were highly correlated across the four, serial, OGTTS, irrespective of the preload conditions (r=0.78–0.87, p<0.0001 for all bivariate comparisons of log-transformed values), yet marked variability was observed between subjects.

## Discussion

Consistent with prior reports [4,10–12], we found that fasting active GLP-1 was higher in men than women. In our cohort, men also had greater peak and AUC results compared to women. These findings are in contrast to another publication which reported that healthy women have higher post-prandial active GLP-1 responses despite lower fasting levels [11]. While lower active GLP-1 secretion has been consistently associated with increasing BMI, our results did not reach statistical significance, which is likely due to the relatively narrow range of BMI among our study participants.

High intra-individual reproducibility suggests that GLP-1 secretion is highly regulated and may be a useful indicator of pharmacologic treatment efficacy, as well as patient compliance with treatment regimens. Stimulated glucose and insulin concentrations are frequently used to determine pharmacologic type 2 diabetes treatment outcomes [13], yet marked intra-individual variability in OGTT results across repeated tests is well-established [14]. Given the relative stability of active GLP-1 responses, measuring GLP-1 in addition may better inform about GLP-1-based pharmacologic interventions.

Prior studies have frequently examined total GLP-1 [3,10,11] and focused specifically on individuals with overweight/obesity [11], prediabetes [3,10,11], and/or type 2 diabetes [10]. Our findings add to the limited body of evidence examining active GLP-1 response in healthy volunteers. Identification of additional factors contributing to inter-individual differences in active GLP-1 and elucidation of physiologic mechanisms driving this variability will be important to better understand the clinical relevance of these findings.

The repeated measures, same-subject crossover design was a key strength of our analysis and provided the opportunity to examine active GLP-1 response both between-subjects and within the same individuals across four serial time points. A key limitation is that our study was not primarily designed to analyze GLP-1 variability.

## Conclusion

In summary, active GLP-1 is higher in men than women and intra-individual reproducibility of active GLP-1 is high, indicating tight regulation. These results will inform the design of future clinical research studies investigating the role of GLP-1 in treatment of obesity and cardiometabolic disease and provide support for potential measurement of GLP-1 as an indicator of treatment efficacy and/or compliance.

## Figures and Tables

**Table 1.** Characteristics of Study Participants, Active GLP-1 Concentrations, and Ratios of Between-subject to Within-subject Variability in Active GLP-1, Overall and by Sex (n=61)

Variable^1^	All All Participants Mean (± SD)	Male	Female
**n**	61	28	33
**Race (n, %)**			
**White/Caucasian**	33, 54%	15, 54%	18
**Black/African American**	20, 33%	9, 32%	11
**Asian**	6, 10%	3, 11%	3
**Other**	2, 3%	1, 3%	1
**Ethnicity**	95% Non-Hispanic	96% Non-Hispanic	94% Non-Hispanic
**Age (years)**	28.3 ± 7.1	29.4 ± 8.1	27.4 ± 6.1
**BMI (kg/m** ^**2**^ **)**	25.9 ± 5.5	27.1 ± 4.5	25.0 ± 6.1
**BMI (kg/m** ^**2**^ **), median (25%, 75%)**	24.9 (21.9, 28.8)	25.9 (24.0, 30.6)	23.1 (20.9, 26.1)
**Fasting Glucose (mmol/L)**	4.7 ± 0.3	4.8 ± 0.3	4.6 ±0.3
**Basal GLP-1 (pmol/L)**	4.8 ± 5.1	6.6 ± 6.1	3.3 ± 3.4*
**Basal GLP-1 (pmol/L), median (25%, 75%)**	2.7 (1.2, 7.6)	5.7 (1.3, 8.6)	2.6 (1.0, 3.5)
**Peak GLP-1 (pmol/L)**	15.8 ± 10.3	18.9 ± 11.9	13.1 ± 8.4*
**Peak GLP-1 (pmol/L), median (25%, 75%)**	13.4 (8.4, 19.7)	18.7 (9.2, 25.2)	12.3 (8.0, 15.7)
**Delta GLP-1 (Peak - Baseline) (pmol/L)**	11.0 ± 9.2	12.4 ± 10.8	9.9 ±7.5
**Delta GLP-1 (pmol/L), median (25%, 75%)**	8.0 (5.4, 13.7)	9.9 (5.3, 17.0)	7.4 (5.4, 12.5)
**GLP-1 AUC (pmol/L/120 min)**	1180.7 ± 806.2	1455.0 ± 970.3	947.9 ± 550.0
**GLP-1 AUC, median (25%, 75%)**	905.3 (599.0, 1566.0)	1226.3 (593.9, 2009.9)	815.8 (639.4, 1139.0)
**γ Ratio** ^2^ **, basal GLP-1 (95% CI)**	4.2 (2.7, 6.7)	5.0 (2.7, 10.2)	3.04 (1.7, 5.9)
**γ Ratio** ^2^ **, peak GLP-1 (95% CI)**	2.4 (1.5, 3.9)	4.3 (2.3, 8.7)	1.3 (0.6, 2.5)
**γ Ratio** ^2^ **, GLP-1 AUC (95% CI)**	3.5 (2.3, 5.6)	3.1 (1.6, 6.3)	3.6 (2.0, 6.9)
**γ Ratio** ^2^ **, Delta GLP-1 (95% CI)**	1.9 (1.2, 3.1)	3.0 (1.6, 6.2)	1.2 (0.6, 2.5)

(GLP-1=glucagon-like-peptide 1

* indicates p-value < 0.05

1 All values expressed as mean ± SD unless otherwise noted

2 γ estimated using imbalanced data (Burdick & Graybill, 1992; Harville & Fenech, 1985). This measure roughly conveys the odds that variability in each GLP-1 measure overall is due to between-participant variability. Ratios expressed as ratio (95% confidence interval)
